# Supplementary material for: The POU Transcription Factor POU-M2 Regulates Vitellogenin Receptor Gene Expression in the Silkworm, Bombyx mori
Source: Genes (Basel). 2020 Apr 6;11(4):394. doi: 10.3390/genes11040394 (PMC7230888; doi:10.3390/genes11040394)
Supplement: Supplementary file 1 [file genes-11-00394-s001.zip › Supplementary Materials.pdf]

```
-2080  TTTGAGCATT ACAAATCAAA AATATAAAGT ACTCCATTTG GGAAATACTG GAATTCTTCG
-2020  AACACGTTTT AAAGCAGCCA TAATCCTGGG TATAAGTGTC ACGAAAAAGT CATGTGTTTT
-1960  GATGGGTAGG TCAGCTGTTT TTACAATAAG ATTAAAACTT CGATTCCCAT GTAATAAAGC
-1900  ATCTCTCACG AATTCCTTCC TCAAATTAGA ATTACTAGAT AATGACCAAG GTGTTCTGGG
-1840  ATTTTTTTTT TGATAAATTG TGTTTTTTAG AAATTATATT TAAATACGAA TTACATATTG
-1780  ATAAAATGAT GAAATATGAA TAGTATTTTT GATCTTACCG ACAGAATAAA AAAAATATGA
-1720  ACTAGTTATT TAAAGAAAAA AATCAAAATT ATCGATAAAG TTGATTATTG AAAGCACGAA
-1660  TAAAACAACA TTTTCTAAAA ATAAATCGTA GCTAGATCGA TTTATCGCCC CCGAAATCCT
-1600  CTGTATACTA AATTTTATGA AAATCGTTTT AGCCGTTTCC GAGATCTTAA TTATATTATT
-1540  TATATTTATA TACAAGAATT GCTCGTTTAA AGACGTAAGA TAAAATACGA TAATAGACTA
-1480  ACATAAATAT ATTAAATATT AATTAAATAC AATAAATGTT ATGAATTAAT AAATTTTCGT  POU
-1420  AATCAATGGC GGTAGATAGT TCTGTAATGC GACCGCGCGT GGGTACATGC CTATTTTTGA
-1360  GCGAATCACG CGAACAAGAT TTATTTGTCT CATATCTCAA AGTAAATGTT ATATGAGGGC
-1300  TGCTGGTCCG CCATGTAGCT GGTTCGATAC TAAACTGAAT AACAAACAAA AAAAACTGGT
-1240  CGTGTAATTT TTAGTGTATT ATCGGTGTTC TGTCTATTGG TGACGTAATT CTGTACGGTT
-1180  TAGGTTTTTT TTGTTTAGAT GGGTGGACGA GCTCACAGCC CACGTGGTGT TAAGTGGTTA
-1120  CTGGAGCCCA TAGACATCTA CAACGTAAAT GCGCCACCCA CCTCGAGATA TAAGTTCTAA  DSX
-1060  GGTCTCAAGT ATAGTTACAA CGGCTGCCCC ACCCTTCAAA CCGAAACGCA TGACTGCTTC
-1000  ACGGCTGAAA TAGGCAGGGT ACCTACCCGT GCGGACTCAC AAGAGGTCCT GCCACCAGTA
 -940  ATCACTAGTA ATAGGTACAA TATGTTAATT GAGTACATTA AAATATGTTT TGAGTTTATC
 -880  TGCAGTATAT TTATTGTGTA ATTAAAAGTA CTGTTATTTT CAAAAATATA AAAAAAGTAT
 -820  ATCATCATCT TGGGCTTTCT TAGTACAACG GATCTTAATT TGCGGTTAGA TGATTAGACA
 -760  AAGATATCTA TATGTCTGCG ACGCATCGCT TATAGCAAAT AGCTTGAAGT CGTCGTGGCC
 -700  TAAAGGATAA GGCGTCCGGT GCATTCGTAT CTAGCGATGC AACGGTGTTC GAATACGGCA
 -640  GGCGGGTACA AATTTTTCTA ATGAAATACG TACTTAACAA ATGTTCTCGA TTGACTTCCA  POU
 -580  CGGTGAAGGA ATATACATCG TGTAATAAAA ATCAAACTCG CAAAGTTATA ATTTGCGTAA  POU
 -520  TTACTGGTGG TAGGTCCTCT TGTGAGTCCG CACGGGTAGG TACCACTGTC CCTGTCTATT
 -460  TCTGCCGTGA AGCAGTAATG CGTTGCGGCT TGAAGGGTGG GGCAGCCGTT AAAAATGGAG
 -400  ACCTTAGAAC CATTATGTTA AGGTAGGTAG TGGCATTTGC GTTGTAGTCT ATGGGCCCCG
 -340  GTAACCACTT GAGGGAGTTT ATATAGAGTC CCTTCTTTAA TTCGCCGTAA AGTCGTGTTT
 -280  TATGTAATTA ACACCACAGT TGAAACAATA TACATTTTAT TGTTGTAGCG GCATAAACAA  POU
 -220  TTCTAAGTAA TTATATTAAA TTAGTTAAAC ATATTATAAA TTATTTTTAC GTTTTAAATT  POU
 -160  TTTGGCGGTA AAAAGGTCTC GTGTCACAGA CTATATTCTG TTTCCCACAT CTCGTGCGTT  E74
 -100  CCGGCTTACG CATCATAAAA AGGTGCGTAC TTAAACGCCA CACACTTAAC CAATAAAATA  POU
  -40  AATAATATAT AACGCTTGAT CAAATACATT TCAGATCAGA ATG
                                                -1
```

Figure S1. Upstream region sequence (promoter) of the cloned *BmVgR* gene. Nucleotides are numbered relative to the translation start site (+1). Putative binding motifs of DNA response elements are shown in color.

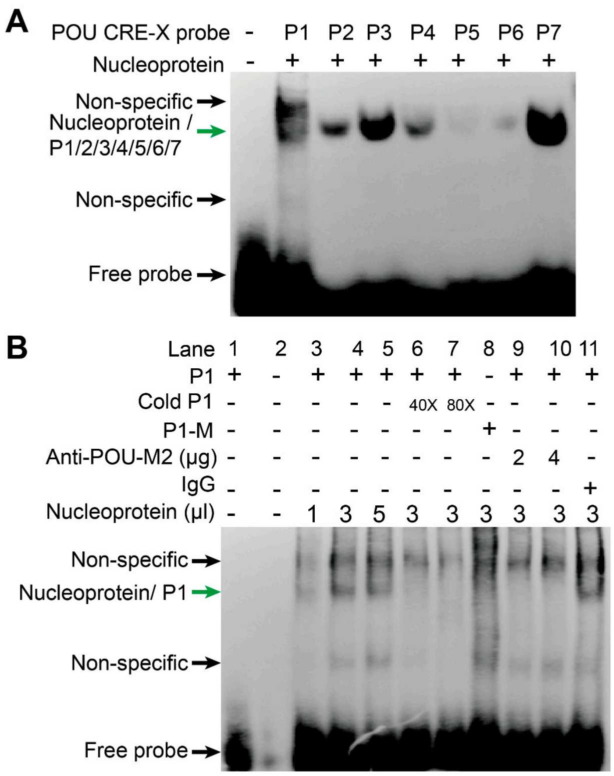

Figure S2. EMSA assays performed to evaluate the binding of POU-specific probes to nucleoprotein of BmNs cells. (A) Binding of the seven POU element probes (P1/P2/P3/P4/P5/P6/P7) to nucleoproteins of POU-M2-overexpressing BmNs cells. (B) Binding of POU CRE-1 to nucleoproteins with cold probe, mutant probe, anti-POU-M2 antibodies, and rabbit IgG.

Table S1 The primers and probes used for this study

Table S2 Predicted CREs in the VgR promoter of six insects by MatInspector.

Table S3 Predicted POU CREs in BmVgR promoter by JASPAR.
